# Noninvasive Coronary Artery Disease Detection Using Retinal Images: A Multimodal Study

**DOI:** 10.1016/j.jacadv.2025.102341

**Published:** 2025-11-19

**Authors:** Xiaohui Li, Xiaoyu Dong, Leilei Chen, Na Su, Kun Huang, Shuo Li, Yuchen Wen, Mingming Zhang, Bing Xu, Songtao Yuan, Junhong Wang, Qiang Chen

**Affiliations:** a School of Computer Science and Engineering, Nanjing University of Science and Technology, Nanjing, Jiangsu, China; b Department of Cardiology, The First Affiliated Hospital of Nanjing Medical University, Nanjing, Jiangsu, China; c Department of Ophthalmology, The First Affiliated Hospital of Nanjing Medical University, Nanjing, Jiangsu, China; d Department of Cardiology, Northern Jiangsu People's Hospital, Yangzhou, China; e Department of Cardiology, People's Hospital of Qijiang District, Chongqing, China; f Department of Cardiology, Liyang People's Hospital, Liyang, China

**Keywords:** coronary artery disease, multimodal feature fusion, noninvasive, retinal imaging

## Abstract

**Background:**

Coronary artery disease (CAD) is a leading cause of morbidity and mortality globally. However, current detection methods have various safety concerns and are not suitable for all populations. Exploring safe, noninvasive detection methods is crucial.

**Objectives:**

The objective of the study was to develop a deep learning-based visual and multimodal detection framework for CAD using retinal images.

**Methods:**

We conducted a multicenter cross-sectional study including 383 patients who underwent successful coronary angiography between November 2022 and September 2024 at 4 hospitals. Three models were developed for CAD detection: a convolutional network-based model for retinal images, a hybrid model combining a medical large language model and multilayer perceptron for clinical indicators, and a multimodal model integrating both via a cross-modal attention mechanism.

**Results:**

The visual algorithm trained solely on retinal images achieved an area under the receiver operating characteristic curve (AUC) of 0.80 (95% CI: 0.75-0.85), with 90.5% sensitivity and 59.6% specificity. Compared to the CAD consortium clinical score, it showed higher accuracy (76.2%) and sensitivity (87.4%) in the test group. Notably, in the intermediate-risk population (clinical score 15%-85%), it outperformed the clinical indicators-only model with higher AUC (0.79 vs 0.75), accuracy, and sensitivity. Multimodal models combining retinal images and clinical indicators further improved detection, with the best model achieving an AUC of 0.91 (95% CI: 0.88-0.94), 87.0% accuracy, and 92.1% sensitivity.

**Conclusions:**

Our findings suggest that retinal images and vasculature may provide useful information for CAD detection. Retinal imaging offers a noninvasive clinical risk factor and holds promise for developing new diagnostic tools. (Exploring the Application of AI-based Fundus Imaging in Risk Stratification and Risk Assessment Models for Coronary Heart Disease; ChiCTR2400092720)

According to estimates from the World Health Organization, approximately 17.9 million people die from cardiovascular diseases (CVDs) each year, accounting for about 32% of all global deaths. Notably, over 75% of these deaths occur in low- and middle-income countries.^1^^,^^2^ Among CVDs, coronary artery disease (CAD) remains a prevalent condition. Despite significant advancements in its diagnosis and treatment, CAD continues to be a major contributor to global morbidity and mortality.^3^

Coronary angiography (CAG) is considered the gold standard for assessing the severity of CAD.^4^^,^^5^ In addition, coronary computed tomography angiography is a commonly used diagnostic tool. However, both CAG and coronary computed tomography angiography have limitations due to their associated risks of radiation exposure and procedural complications, which can restrict their practical application.^6^^,^^7^

Recent evidence suggests that the eye, as a unique window, offers a promising noninvasive approach to understanding CAD.^8^ The retinal and choroidal microvasculature is supplied by the ophthalmic artery, a terminal branch of the aorta, similar to the coronary arteries, which are also branches of the aorta. As such, it is hypothesized that both the retinal and choroidal vasculature may be affected by coronary artery stenosis.^9^ Fortunately, the unique anatomy of the eye allows for the visualization of retinal and choroidal vessels using optical coherence tomography (OCT) and OCT angiography (OCTA). These imaging techniques are noninvasive, radiation-free, and do not require the injection of contrast agents. Consequently, the retina provides a window for detecting microvascular changes associated with CAD.

A growing body of research has investigated the association between retinal/choroidal vasculature and CAD.^10^, ^11^, ^12^, ^13^, ^14^ Notably, OCTA-derived blood flow parameters have been identified as independent risk factors for major adverse cardiovascular events in CAD.^15^ However, current research linking the retina to CAD remains largely at the statistical analysis stage, limiting its practical application in clinical settings. With the advancement of artificial intelligence, deep learning algorithms have emerged as promising tools for disease diagnosis and prediction. Therefore, this study aimed to leverage deep learning algorithms to explore the potential of OCT/OCTA imaging in predicting CAD. Furthermore, we propose to integrate multitype clinical data to develop a multimodal, rapid diagnostic model for CAD.

## Methods

### Participants

This multicenter cross-sectional study enrolled patients who underwent successful CAG between November 2022 and September 2024 at 4 hospitals: the First Affiliated Hospital of Nanjing Medical University, the Northern Jiangsu People's Hospital, People's Hospital of Qijiang District, and Liyang People's Hospital. CAD was defined as more than 50% narrowing in any major coronary artery, including the left main, left anterior descending, left circumflex, or right coronary artery, as determined by CAG. Patients were not selected in advance to undergo CAG for the purpose of this study, thereby avoiding potential ethical concerns. After identifying patients who had completed CAG during this period, we applied the predefined inclusion and exclusion criteria. Eligible patients were then invited to undergo retinal imaging (OCT/OCTA) as part of the study. The imaging procedures were performed by professionally trained cardiologists and ophthalmologists. Inclusion criteria included the following: 1) age ≥18 years; 2) successful completion of CAG; and 3) completion of OCT/OCTA imaging. Exclusion criteria included the following: 1) patients with retinal diseases (eg, diabetic retinopathy, diabetic macular edema, vitreomacular traction, age-related macular degeneration); 2) history of cataract surgery within the past 3 months, previous laser surgery, intravitreal drug therapy, or major retinal surgery; 3) patients with congenital heart disease, valvular heart disease, pericardial disease, or aortic dissection; and 4) patients diagnosed with malignant tumors. All eligible patients provided informed consent before undergoing CAG, agreeing to participate in the study and to allow the use of their data for research purposes.

Following enrollment, baseline interviews were conducted to collect data on lifestyle factors (eg, smoking), clinical pain symptoms, medical history, and medications. Additional demographic characteristics, risk factors, laboratory test, and electrocardiographic were extracted from medical records postprocedure. Table 1 summarizes part of the key clinical characteristics. Standardized imaging protocols were employed to perform 3-dimensional OCT imaging of the macular region in both eyes of each patient.

**Table 1:** Clinical Characteristics

	Control Group (n = 165)	CAD Group (n = 218)	*P* Value
Baseline			
Age (y)^a^	61.81 ± 11.42 (25-82)	63.44 ± 10.81 (31-83)	0.23
Male, n (%)^b^	76 (46.06)	153 (70.18)	**<0.001**
BMI (kg/m^2^)^a^	25.12 ± 3.73 (16.9-41.6)	24.88 ± 3.43 (17.9-50)	0.81
Medical history			
Hypertension, n (%)^b^	78 (47.27)	157 (72.02)	**<0.001**
Diabetes mellitus, n (%)^b^	29 (17.58)	69 (31.65)	**0.003**
Smoking, n (%)^b^	21 (12.73)	74 (33.94)	**<0.001**
Cerebral infarction, n (%)^b^	9 (5.45)	16 (9.70)	0.60
Heart failure, n (%)^b^	3 (1.82)	1 (0.46)	0.43
Medications			
β-Blockers, n (%)^b^	65 (39.39)	137 (62.84)	**<0.001**
ACEI or ARB, n (%)^b^	38 (23.03)	56 (25.69)	0.63
MRA, n (%)^b^	10 (6.06)	13 (5.96)	1.00
Diuretics, n (%)^b^	33 (20.00)	21 (9.63)	**0.006**
CCB, n (%)^b^	50 (30.30)	73 (33.49)	0.58
Antiplatelet, n (%)^b^	71 (43.03)	182 (83.49)	**<0.001**
Anticoagulant, n (%)^b^	33 (20.00)	93 (42.66)	**<0.001**
Statin, n (%)^b^	120 (72.73)	215 (98.62)	**<0.001**
Insulin, n (%)^b^	1 (0.61)	12 (5.50)	**0.019**
Oral hypoglycemic, n (%)^b^	24 (14.55)	54 (24.77)	**0.020**
GLP-1, n (%)^b^	1 (0.61)	4 (1.83)	0.55

Statistical results are expressed as mean ± SD (min-max) or n (%).

ACRI/ARB = angiotensin-converting enzyme inhibitors or II receptor blockers; BMI = body mass index; CCB = calcium channel blockers; GLP-1 = glucagon-like peptide-1; MRA = mineralocorticoid receptor antagonists; n = number of patients.

A *P* value <0.05 is considered statistically significant. Bold indicate that the characteristic exhibits a significant difference (*P* < 0.05) between the 2 groups (CAD group and Control group).

a Mann-Whitney U test.

b Chi-square test.

### Data processing and model architecture

Details about data processing and model architecture can be found in the eData Processing and Model Architecture in Supplement Appendix.

### Experimental design

We designed 5 types of experiments to better verify the role of retinal images in CAD classification decisions. Metrics such as classification accuracy (ACC), sensitivity (Sn), specificity (Sp), F1-Score, positive predictive value (PPV), negative predictive value (NPV), and the area under the receiver operating characteristic (ROC) curve (AUC) were calculated. For comparison with the widely recognized CAD consortium clinical score, we followed the methods of Lin et al.^16^ Two threshold points were selected from the ROC curve for evaluation: the point with the maximum sum of sensitivity and specificity (max of Sn + Sp) and the point of high-sensitivity (Sn = 80%).

For the routine evaluation, we performed 5-fold cross-validation on the 383 patients, independently repeating the entire process 3 times with different random seeds to ensure robustness and stability. The final results are reported as the mean and SD across all 15 validation runs. In comparison with the CAD consortium, we trained the CAD detection model using data from 233 patients and computed the CAD consortium clinical score as well as the AI model results for the remaining 150 patients.

#### Multimodal data ablation

We conducted comparative experiments using all clinical indicators (45 items) and retinal images (OCT/OCTA/projection maps). Three settings were tested: 1) only 45 clinical indicators; 2) only retinal images; and 3) both clinical indicators and retinal images. This aimed to validate the independent role of retinal images in CAD detection and the improvement in accuracy with multimodal data integration.

#### Simulated diagnostic workflow

Patient clinical indicators were categorized into groups: baseline, clinical pain symptoms, medical history, medication, laboratory tests, and standard electrocardiogram (ECG) parameters (details in Supplemental Table 1). These categories roughly simulate the diagnostic workflow after patient admission. Starting with baseline data, additional clinical indicator groups (baseline, baseline + clinical pain symptoms, baseline + clinical pain symptoms + medical history, …) were progressively added to train the model. Each set of experiments was conducted with and without retinal images, resulting in 12 experimental groups in total. This design aimed to evaluate the role of retinal images at different stages of clinical diagnosis and compare their contribution to CAD detection with specific clinical indicator groups.

#### Univariable analysis

Full clinical indicator sets may be too complex, and some variables might be irrelevant or even detrimental to CAD detection. Therefore, we performed univariable statistical analyses on each indicator and identified 25 variables (Supplemental Table 2) with significant differences (*P* < 0.05) between the control and CAD groups. These 25 variables, combined with retinal images, were used as inputs for CAD classification models.

#### Multivariable analysis

**Figure 1:**
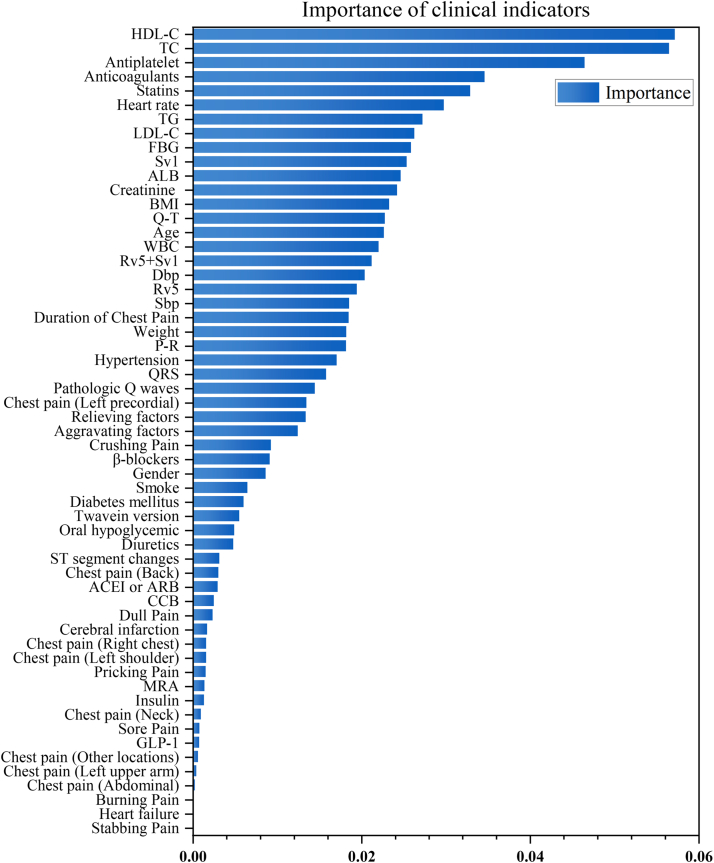
**Importance Ranking of Clinical Indicators Obtained by Random Forest Model** ACRI/ARB = angiotensin-converting enzyme inhibitors or II receptor blockers; ALB = albumin; BMI = body mass index; CCB = calcium channel blockers; DBP = diastolic blood pressure; FBG = fasting blood glucose; GLP-1 = glucagon-like peptide-1; HDL-C = high-density lipoprotein cholesterol; HR = heart rate; LDL-C = low-density lipoprotein cholesterol; MRA = mineralocorticoid receptor antagonists; P-R = P-R interval; QRS = QRS duration; Q-T = QT interval; RV5 = R-wave amplitude in lead V5; SBP = systolic blood pressure; SV1 = S wave amplitude in lead V1; TC = total cholesterol; TG = triglycerides; WBC = white blood count.

We fit a random forest model based on the complete set of 45 clinical indicators for a multifactorial conjoint analysis. The random forest model quantified and ranked the importance (Figure 1) of each clinical indicator in CAD testing. We took the top 15 and top 30 clinical indicators (Supplemental Table 2) in terms of importance and conducted 2 sets of experiments, each of which was divided into 2 groups without and with retinal images, for a total of 4 sets of experiments.

#### Pretest probability

As a recognized standard, we calculated the CAD consortium clinical score based on patient age, sex, clinical pain symptoms, and cardiovascular risk factors, shown in Supplemental Table 2. Based on the clinical indicators used previously, we trained new CAD classification models with the following configurations: clinical indicators only, retinal images only, and combined multimodal data. Following Lin et al.,^16^ we evaluated and compared the models using the points of maximum (Sn + Sp) and high sensitivity (Sn = 80%) from the ROC curve. These comprehensive experimental designs ensured a thorough evaluation of the role of retinal images and their integration with clinical data in CAD detection tasks.

### Statistical analysis

All statistical analyses and model evaluations were performed using Python (v3.9.7; SciPy v1.11.4; Scikit-Learn v1.3.2; PyTorch v1.13.0).

For descriptive statistics, continuous variables are reported as mean ± SD (range: min-max), and categorical variables are summarized as counts and percentages. For univariable group comparisons, the Kolmogorov-Smirnov test was first used to assess normality. If the normality assumption was satisfied, between-group comparisons were conducted using the Student’s t-test; otherwise, the Mann-Whitney U test was applied. Categorical variables were compared using the chi-square test. A *P* < 0.05 was considered statistically significant. For variables with sparse missingness, simple imputation was applied: continuous variables were imputed with the group mean, and categorical variables were imputed with the mode. Variables with extensive (>30%) missingness were excluded from downstream analysis. The variables excluded due to high missingness are listed in Supplemental Table 3.

For model evaluation and reporting, all predictive machine learning models were trained using the PyTorch framework and produced probabilistic outputs for the CAD categories. ROC curves, AUCs, confusion matrices, and threshold-dependent metrics (accuracy, sensitivity, specificity, F1-score, PPV, and NPV) were computed using functions from SciPy and Scikit-Learn. AUCs are reported with 95% CIs. When comparing AUCs between 2 correlated models on the same test set, the DeLong *P* value was reported. A *P* < 0.05 was considered statistically significant.

## Results

### Study cohort

**Figure 2:**
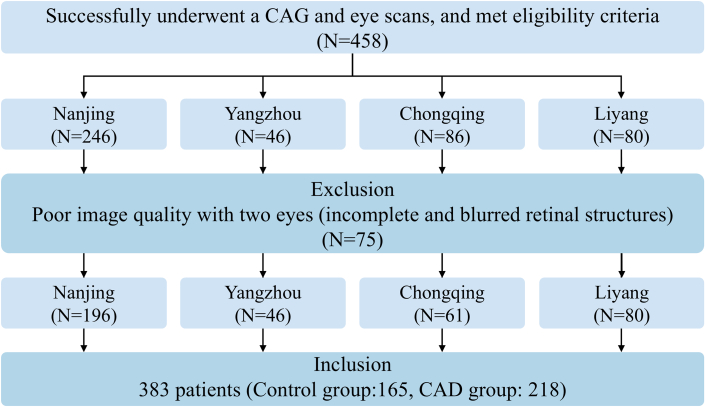
**Inclusion–Exclusion Flowchart of Coronary Artery Disease and Normal Control Cohorts** CAD = coronary artery disease; CAG = coronary angiography.

Between November 2022 and September 2024, a total of 458 patients who met the inclusion criteria were enrolled from 4 research institutions (Figure 2). Seventy-five patients with unqualified retinal images in both eyes were excluded, whereas those with valid retinal images in 1 eye were retained for subsequent analysis. Among the remaining 383 patients, 165 were in the control group and 218 were in the CAD group. Of these, 344 patients had retinal images for both eyes and 39 had retained 1 eye. The clinical characteristics of the enrolled patients, including baseline data, clinical pain symptoms, medical history, medication, laboratory tests, and standard ECG parameters, as well as group statistics, are summarized in Supplemental Table 1.

### Multimodal data ablation

**Figure 3:**
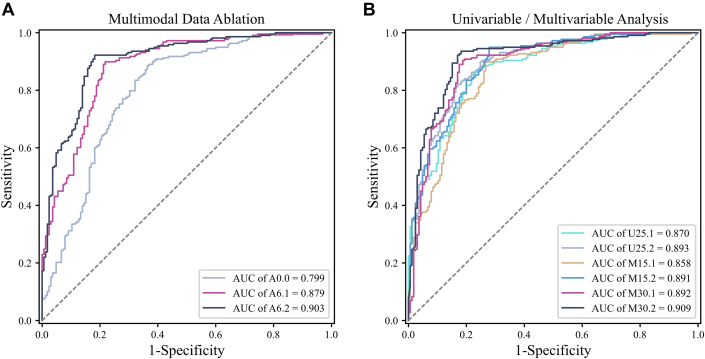
**ROC Curves of Multimodal Data Ablation, Univariable and Multivariable Analysis** (A) Multimodal data ablation. (B) Univariable/multivariable analysis. AUC = area under the receiver operating characteristic curve.

The comparison results of multimodal data experiments, including AUC, accuracy, sensitivity, specificity, F1-score, PPV, and NPV, are shown in Table 2 and Figure 3A. Using only retinal images (A0.0), the deep convolutional neural network algorithm achieved an AUC of 0.80 (95% CI: 0.75-0.85), with an accuracy of 77.2%, and high sensitivity of 90.5%. Using all 45 clinical indicators, the large language mode and multilayer perception algorithm (A6.1) achieved an AUC of 0.88 (95% CI: 0.84-0.91; DeLong *P* = 0.006 VS A0.0), with an accuracy of 84.5%. In addition to sensitivity (89.4% vs 90.5%), the algorithm performed better than the retinal image-only model in terms of specificity, F1-score, PPV, and NPV. When combining retinal image with clinical information, the multimodal CAD detection model (A6.2) achieved an AUC of 0.90 (95% CI: 0.87-0.94; DeLong *P* < 0.001 VS A0.0), achieving the highest performance across all evaluation metrics. However, the difference of AUC between A6.2 and A6.1 was not statistically significant (*P* = 0.28).

**Table 2:** Algorithm Performance of Multimodal Data for Detecting Coronary Artery Disease

Algorithm	Clinical	Image	AUC (95% CI)	Accuracy	Sensitivity	Specificity	F1_Score	PPV	NPV
A0.0		√	0.799 (0.751-0.846)	77.19 ± 0.41	90.53 ± 0.96	59.60 ± 2.13	81.94 ± 0.15	75.04 ± 0.73	82.78 ± 0.83
A6.1∗	√		0.879 (0.844-0.914)	84.50 ± 1.33	89.44 ± 0.45	77.98 ± 2.73	86.80 ± 1.01	84.46 ± 1.70	84.96 ± 0.98
A6.2∗	√	√	0.903 (0.871-0.935)	86.33 ± 1.09	90.69 ± 1.89	80.61 ± 3.64	88.35 ± 0.85	86.36 ± 2.00	86.94 ± 1.81

Results are expressed as mean ± SD.

DeLong statistics: A6.1 vs A0.0 (*P* = 0.006); A6.2 vs A0.0 (*P* < 0.001); A6.2 vs A6.1 (*P* = 0.28).

AUC = area under the receiver operating characteristic curve; NPV = negative predictive value; PPV = positive predictive value.

∗ Indicates statistically significant. See Delong statistics for *P* values.

### Simulated diagnostic workflow

The experimental results are shown in Table 3. When only baseline data (sex, age, body mass index, systolic blood pressure, and diastolic blood pressure) from patients were used (A1.1), the algorithm's AUC was 0.66 (95% CI: 0.60-0.72), which was significantly lower (*P* < 0.001) than the retinal image-based algorithm (A0.0: 0.80; 95% CI: 0.75-0.85). The inclusion of retinal images (A1.2) significantly (vs A1.1, *P* < 0.001) improved the algorithm’s AUC to 0.81 (95% CI: 0.77-0.86), with a sensitivity of 91.7%. As additional clinical information were added, it was observed that even when baseline data, clinical pain symptoms, and medical history were input (A3.1), the algorithm's performance still did not reach the level of the retinal image-based (A0.0) model (AUC: 0.75 vs 0.80, accuracy: 74.6% vs 77.2%).

**Table 3:** Algorithm Performance of Simulated Diagnostic Workflow

	C1	C2	C3	C4	C5	C6	Img	AUC (95% CI)	Accuracy	Sensitivity	Specificity	F1_Score	PPV	NPV
A1.1	√							0.659 (0.603-0.715)	67.90 ± 0.52	80.84 ± 3.88	50.71 ± 4.63	74.02 ± 1.08	69.32 ± 1.34	50.71 ± 4.63
A1.2∗	√						√	0.811 (0.766-0.857)	78.41 ± 0.83	91.72 ± 0.91	61.41 ± 3.11	82.83 ± 0.40	75.96 ± 1.32	84.33 ± 0.43
A2.1	√	√						0.729 (0.677-0.780)	72.16 ± 0.92	76.46 ± 2.26	66.46 ± 1.95	75.65 ± 1.20	75.54 ± 0.43	68.74 ± 1.37
A2.2∗	√	√					√	0.816 (0.772-0.861)	78.41 ± 1.78	90.37 ± 0.78	62.63 ± 3.94	82.70 ± 1.22	76.45 ± 2.03	83.42 ± 1.62
A3.1	√	√	√					0.752 (0.701-0.802)	74.59 ± 0.40	75.85 ± 0.72	72.93 ± 0.93	77.16 ± 0.41	79.41 ± 0.41	70.13 ± 0.77
A3.2∗	√	√	√				√	0.854 (0.815-0.894)	81.20 ± 0.45	88.54 ± 1.83	71.52 ± 2.64	84.26 ± 0.41	80.64 ± 1.14	83.10 ± 2.11
A4.1	√	√	√	√				0.855 (0.817-0.893)	81.12 ± 1.05	85.01 ± 2.31	75.96 ± 4.13	83.62 ± 0.81	82.55 ± 2.28	79.78 ± 1.71
A4.2	√	√	√	√			√	0.876 (0.839-0.913)	84.08 ± 0.52	90.53 ± 1.30	75.56 ± 0.93	86.61 ± 0.55	83.11 ± 0.48	86.05 ± 1.58
A5.1	√	√	√	√	√			0.865 (0.827-0.902)	82.68 ± 0.84	85.65 ± 2.49	78.79 ± 5.28	84.91 ± 0.19	84.65 ± 2.76	81.15 ± 1.77
A5.2	√	√	√	√	√		√	0.886 (0.851-0.920)	84.60 ± 0.52	90.84 ± 0.45	76.36 ± 0.61	86.97 ± 0.46	83.60 ± 0.39	87.02 ± 0.39
A6.1	√	√	√	√	√	√		0.879 (0.844-0.914)	84.50 ± 1.33	89.44 ± 0.45	77.98 ± 2.73	86.80 ± 1.01	84.46 ± 1.70	84.96 ± 0.98
A6.2	√	√	√	√	√	√	√	0.903 (0.872-0.935)	86.33 ± 1.09	90.69 ± 1.89	80.61 ± 3.64	88.35 ± 0.85	86.36 ± 2.00	86.94 ± 1.81

C1 = baseline; C2 = clinical pain symptoms; C3 = medical history; C4 = medications; C5 = laboratory test; C6 = electrocardiography.

Abbreviations as in Table 2.

DeLong statistics: A1.2 vs A1.1 (*P* < 0.001); A2.2 vs A2.1 (*P* = 0.015); A3.2 vs A3.1 (*P* = 0.003); A4.2 vs A4.1 (*P* = 0.94); A5.2 vs A5.1 (*P* = 0.30); A6.2 vs A6.1 (*P* = 0.18).

∗ Indicates statistically significant. See Delong statistics for *P* values.

Comparing algorithms A3.1, A3.2, and A4.1, the addition of retinal images and patient medication history showed a similar improvement in CAD detection performance (AUC: 0.85 vs 0.86), but the inclusion of retinal images led to a higher sensitivity (88.5% vs 85.0%). Comparing algorithms A4.1, A4.2, and A6.1, the single-modality algorithm with the addition of laboratory tests and ECG achieved an AUC of 0.88 (95% CI: 0.84-0.91) with an accuracy of 84.5%, whereas the multimodal algorithm with retinal images yielded similar results, with an AUC of 0.88 (95% CI: 0.84-0.91) and an accuracy of 84.1%.

In pairwise comparisons (A1.1 vs A1.2, A2.1 vs A2.2, among others), the inclusion of retinal images consistently improved CAD detection performance to varying degrees. Statistically significant (*P* < 0.001, *P* = 0.015, *P* = 0.003) improvements were observed in the first 3 comparisons (A1.1 vs A1.2, A2.1 vs A2.2, and A3.1 vs A3.2). In contrast, no statistically significant differences (*P* = 0.94, *P* = 0.30, *P* = 0.18) were observed in the latter 3 comparisons (A4.1 vs A4.2, A5.1 vs A5.2, and A6.1 vs A6.2). Notably, retinal images enabled the algorithm to maintain a high sensitivity above 90% in 5 of 6 comparisons, with the exception of A3.2, which still achieved a relatively high sensitivity of 88.3%.

### Univariable analysis

The results of the experiments are shown in Table 4 and Figure 3B. Using these indicators, the algorithm based solely on clinical information (U25.1) achieved an AUC of 0.87 (95% CI: 0.83-0.91) and an accuracy of 83.2%. The algorithm's overall performance was slightly lower than that of the model using all 45 clinical indicators (A6.1). The multimodal algorithm (U25.2), which combined retinal images and clinical information, achieved a higher AUC (0.89 vs 0.87), but with a slight decrease in sensitivity (88.8% vs 89.5%), and there was no significant difference in AUC compared to U25.1 (*P* = 0.48).

**Table 4:** Algorithm Performance of Univariable Analysis

Algorithm	Clinical	Image	AUC (95% CI)	Accuracy	Sensitivity	Specificity	F1_Score	PPV	NPV
U25.1	√		0.870 (0.832-0.907)	83.21 ± 0.91	89.45 ± 3.73	74.95 ± 2.99	85.75 ± 1.20	82.74 ± 1.25	85.33 ± 3.91
U25.2	√	√	0.893 (0.860-0.926)	84.33 ± 0.94	88.84 ± 1.16	78.38 ± 3.65	86.59 ± 0.53	84.68 ± 2.05	84.48 ± 0.59

DeLong statistics: U25.2 vs U25.1 (*P* = 0.48).

Abbreviations as in Table 2.

### Multivariable analysis

The results are shown in Table 5 and Figure 3B. Comparing algorithms M15.2 and U25.1, the 15 clinical indicators selected through multivariable analysis achieved similar CAD detection performance as the 25 clinical indicators selected through univariable analysis (AUC: 0.89 vs 0.89; accuracy: 84.2% vs 84.3%), but the algorithm M15.2 exhibited higher sensitivity (93.3% vs 88.8%). Furthermore, algorithms M30.1 and M30.2, using the top 30 clinical indicators, outperformed the model using all clinical indicators (A6.1 and A6.2), with M30.2 achieving the highest AUC value of 0.91 (95% CI: 0.88-0.94) and an accuracy of 87.0%. The inclusion of retinal images enabled the CAD detection algorithm to maintain a high level of sensitivity (>92.0%).

**Table 5:** Algorithm Performance of Multivariable Analysis

Algorithm	Clinical	Image	AUC (95% CI)	Accuracy	Sensitivity	Specificity	F1_Score	PPV	NPV
M15.1	√		0.858 (0.821-0.896)	81.45 ± 2.04	89.27 ± 2.82	71.11 ± 1.53	84.52 ± 1.88	80.58 ± 1.18	84.60 ± 3.17
M15.2	√	√	0.891 (0.859-0.923)	84.24 ± 0.92	93.27 ± 1.39	71.72 ± 2.45	87.12 ± 0.71	81.43 ± 1.22	89.77 ± 1.98
M30.1	√		0.892 (0.859-0.926)	85.55 ± 0.55	89.28 ± 1.90	80.61 ± 1.60	87.53 ± 0.60	86.00 ± 0.85	85.26 ± 2.16
M30.2	√	√	0.909 (0.878-0.941)	86.95 ± 1.20	92.05 ± 0.95	80.20 ± 1.53	88.88 ± 1.02	86.05 ± 1.07	88.97 ± 1.32

DeLong statistics: M15.2 vs M15.1 (*P* = 0.22); M30.2 vs M30.1 (*P* = 0.43).

Abbreviations as in Table 2.

### Pretest probability

**Figure 4:**
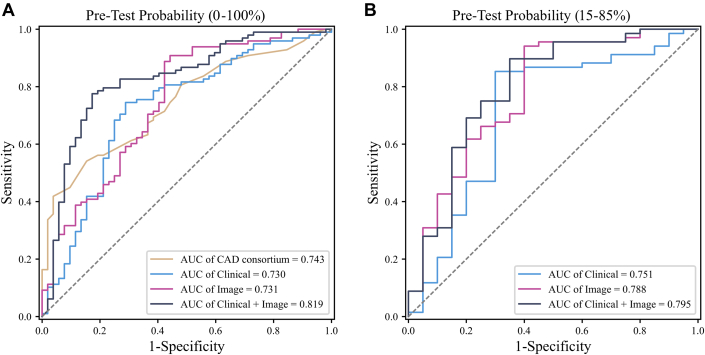
**Receiver Operating Characteristic Curves of Pretest Probability** (A) Pretest probability (0%-100%). (B) pretest probability (15%-85%). Abbreviations as in Figures 2 and 3.

The results are shown in Table 6 and Figure 4A. Using the operating point with the maximum sum of sensitivity and specificity, the retinal image-only algorithm (P1.3) achieved a sensitivity of 87.4% and a specificity of 55.1% in the test group. The multimodal algorithm (P1.4), combining clinical and image data, achieved a sensitivity of 74.2% and specificity of 80.8%. Although the overall results were better than the CAD consortium score, the improvement was not statistically significant (*P* = 0.14). Using the higher-sensitivity operating point (Sn = 80%), the retinal image-only algorithm (P2.3) had a sensitivity of 78.6% and specificity of 59.0%, whereas the multimodal algorithm (P2.4) achieved a sensitivity of 79.3% and specificity of 73.2%.

**Table 6:** Comparison of AI Algorithms and CAD Consortium Clinical Score

Algorithm	Methods	AUC (95% CI)	Accuracy	Sensitivity	Specificity	F1_Score	PPV	NPV
	Max Spe + Sen							
P1.1	CAD consortium clinical score	0.743 (0.665-0.821)	64.67	54.08	84.62	66.67	86.89	49.44
P1.2	AI algorithm (clinical)	0.730 (0.646-0.813)	70.67	67.35	76.92	74.89	84.73	55.84
P1.3	AI algorithm (image)	0.731 (0.644-0.819)	76.22	87.41	55.13	82.76	78.61	70.07
P1.4	AI algorithm (Clinical + Image)	0.819 (0.746-0.891)	76.44	74.15	80.77	80.38	88.20	62.61
	Sen = 80%							
P2.1	CAD consortium clinical score	0.743 (0.665-0.821)	70.67	80.61	51.92	78.22	75.96	58.70
P2.2	AI algorithm (clinical)	0.730 (0.646-0.813)	69.78	79.59	51.28	77.51	75.58	56.87
P2.3	AI algorithm (image)	0.731 (0.644-0.819)	71.78	78.57	58.97	78.41	78.29	59.55
P2.4	AI algorithm (clinical + image)	0.819 (0.746-0.891)	77.33	79.25	73.72	82.06	85.10	65.28

Prevalence of CAD (one coronary lesion ≥50%) in the test group was 65.3% (98/150).

DeLong statistics: P1.2 vs P1.1 (*P* = 0.76); P1.3 vs P1.1 (*P* = 0.98); P1.4 vs P1.1 (*P* = 0.14).

AI = artificial intelligence; CAD = coronary artery disease; Max Spe + Sen = maximum sum of sensitivity and specificity; other abbreviations as in Table 2.

In the test group, for patients with a pretest probability in the moderate range (15% - 85%), retinal images showed an advantage over clinical indicators, as shown in Table 7 and Figure 4B. The AUC (P3.1) was 0.79 (95% CI: 0.67-0.91), with accuracy and sensitivity of 83.7% and 94.1%, and PPV and NPV of 86.2% and 71.9%, respectively. Although the multimodal algorithm (P3.3) achieved a better AUC (0.80), its accuracy and sensitivity were lower than the retinal image-only algorithm (83.0% vs 83.7%, 89.2% vs 94.1%). No significant differences were observed among the 3 experiments in pairwise comparisons.

**Table 7:** Comparison of the AI Algorithm With CAD Consortium Clinical Scores in Intermediate-Risk Population (15%-85%)

Algorithm	Clinical	Image	AUC (95% CI)	Accuracy	Sensitivity	Specificity	F1_Score	PPV	NPV
P3.1		√	0.788 (0.669-0.907)	83.71 ± 1.31	94.12 ± 2.94	48.33 ± 11.55	89.93 ± 0.79	86.18 ± 2.29	71.94 ± 6.65
P3.2	√		0.751 (0.624-0.878)	77.27 ± 1.14	82.84 ± 3.70	58.33 ± 11.55	84.91 ± 1.03	87.23 ± 2.56	50.05 ± 1.93
P3.3	√	√	0.795 (0.664-0.927)	82.95 ± 1.97	89.22 ± 2.25	61.67 ± 2.89	88.99 ± 1.37	88.78 ± 0.84	62.94 ± 6.09

DeLong statistics: P3.2 vs P3.1 (*P* = 0.49); P3.3 vs P3.1 (*P* = 0.87); P3.3 vs P3.2 (*P* = 0.40).

Abbreviations as in Table 2 and 6.

## Discussion

In this large-scale multicenter cross-sectional study, we developed a multimodal deep learning model for the detection of CAD based on data collected from 383 patients across 4 hospitals. The model integrates 2 networks using different modalities: retinal images (OCT/OCTA/projection map) and clinical indicators. A deep convolutional neural network was constructed as a feature extractor for retinal images, whereas a multilayer perception combined with a large language mode was developed as a textual perceiver for clinical indicators. Extensive experiments on the internal data set validated the detection performance of the multimodal model. The best experimental results achieved an AUC of 0.91 (95% CI: 0.88-0.94), an accuracy of 87.0%, and a sensitivity of 92.1%. These findings demonstrate the feasibility of using retinal images for CAD detection tasks.

In previous studies exploring the relationship between retinal images and CVDs, Poplin et al.^17^ demonstrated that vascular regions in the retina are associated with cardiovascular risk factors, developing a visual model based on fundus photography with an AUC of 0.70 (95% CI: 0.65-0.74). Similarly, Lee et al.^18^ identified the role of the optic disc and vessels in fundus images for predicting CVD, achieving an AUC of 0.69 (95% CI: 0.67-0.70) with DenseNet-169. These studies highlight the need for further research to improve predictive performance. In our study, the proposed purely vision-based deep learning algorithm for CAD detection performed well, as shown in Table 2. In 5-fold cross-validation, the algorithm (A0.0) achieved an AUC of 0.80 (95% CI: 0.75-0.85), a sensitivity of 90.5%, and a specificity of 59.6%. In addition, compared with the CAD consortium clinical score (Table 6, P1.1), which uses clinical indicators listed in Supplementary Table 2, our algorithm (P1.3) achieved slightly lower AUC in the test set (0.73 vs 0.74) but demonstrated higher accuracy (76.2% vs 64.7%) and sensitivity (87.4% vs 54.1%). The ROC curve, shown in Figure 4A, reveals that the high AUC of the CAD consortium clinical score is primarily attributed to its high specificity (84.6%), but it has lower recognition capability for CAD patients. For disease screening tasks, where high sensitivity is essential to identify all affected patients, our retinal image-based algorithm has an advantage. Further analysis shows that, based on the CAD consortium clinical score, the model trained solely on retinal images (P3.1) achieved higher AUC (0.79 vs 0.75), accuracy, sensitivity, and F1-score compared to the model trained on clinical indicators (P3.2) in the intermediate risk group (15% - 85%) of test patient, as shown in Table 7. Although the difference was not statistically significant (*P* = 0.49), these results provide additional evidence supporting the feasibility of using retinal images for CAD detection.

Clinical indicators such as age, sex, diabetes, hypertension, total cholesterol, and high-density lipoprotein cholesterol are well-known cardiovascular risk factors.^18^ Our experimental results are consistent with this consensus. A parametric model using all 45 clinical indicators (A6.1) achieved an AUC of 0.88 (95% CI: 0.84-0.91) and a sensitivity of 89.4%. In the evaluation of simulated diagnostic workflows, models with a small subset of clinical indicators (A1.1, A2.1, A3.1) performed worse than the retinal image-based algorithm (A0.0). Only when sufficient clinical information was included did models (A4.1, A5.1, A6.1) outperform the vision-based algorithm. This is reasonable, as clinical information is more directly associated with CVD. These results suggest that retinal imaging may serve as a valuable complementary modality for CAD detection, particularly in settings where noninvasive risk stratification tools are needed. In addition, statistically significant improvements were observed in the first 3 comparisons (A1.1 vs A1.2, A2.1 vs A2.2, and A3.1 vs A3.2), indicating that retinal images provided added diagnostic value when the clinical model included only baseline data, pain symptoms, and medical history. In contrast, no statistically significant differences were observed in the latter 3 comparisons (A4.1 vs A4.2, A5.1 vs A5.2, and A6.1 vs A6.2), suggesting that once medications, laboratory results, or ECGs were included, the incremental benefit of retinal images diminished. These findings underscore that the diagnostic contribution of retinal imaging may be most pronounced in earlier, less information-rich stages of the diagnostic workflow. Notably, the performance of algorithm A4.2 was comparable to A6.1, suggesting that when essential clinical information is available, retinal images may provide diagnostic value that complements traditional assessments such as laboratory tests and ECGs.

Al-Zaiti et al.^19^ demonstrated an AUC of 0.91 (95% CI: 0.87-0.96) in detecting complete coronary artery occlusion using a small set of selected key features, suggesting that not all clinical indicators are equally useful for disease detection. Similarly, we conducted related experiments in our study. Through univariable analysis, we identified 25 clinical indicators with significant differences between the control and CAD groups. The performance of algorithm U25.1, which used these 25 indicators, was only slightly inferior to that of algorithm A6.1, which used all 45 indicators. Moreover, using feature importance derived from a random forest model, algorithm M30.1, which used the top 30 clinical indicators, outperformed algorithm A6.1 in detection performance. Our findings suggest that certain clinical indicators may negatively affect CAD detection tasks (depending on the specific task). Notably, in the 30 indicators selected via multivariable analysis, traditional cardiovascular risk factors such as sex, smoking, and diabetes were absent, despite their significance in univariable analysis. These results highlight the importance of feature selection engineering in future CAD or CVD detection tasks.

In addition, we presented the results of multimodal models trained on retinal images combined with clinical indicators. Our findings indicate that retinal images may serve as useful risk factors for diagnosing CAD (Central Illustration), and in several experimental settings, multimodal CAD detection models outperformed unimodal models, with some differences reaching statistical significance (Table 2, Table 3, Table 4, Table 5, Table 6). Similar conclusions were reached by Poplin et al.^17^ and Lee et al.^18^ In cases where clinical information is limited, retinal images help maintain multimodal model performance comparable to purely vision-based models. When sufficient clinical information is available, the inclusion of retinal images consistently contributes to stable or improved detection performance, highlighting their complementary value in CAD assessment. Notably, in most models incorporating retinal images, whether unimodal or multimodal, higher sensitivity was consistently observed, suggesting a potential role of retinal images in identifying CAD patients. The confusion matrices for all algorithms are provided in Supplemental Figures 4 and 5.

**Central Illustration:**
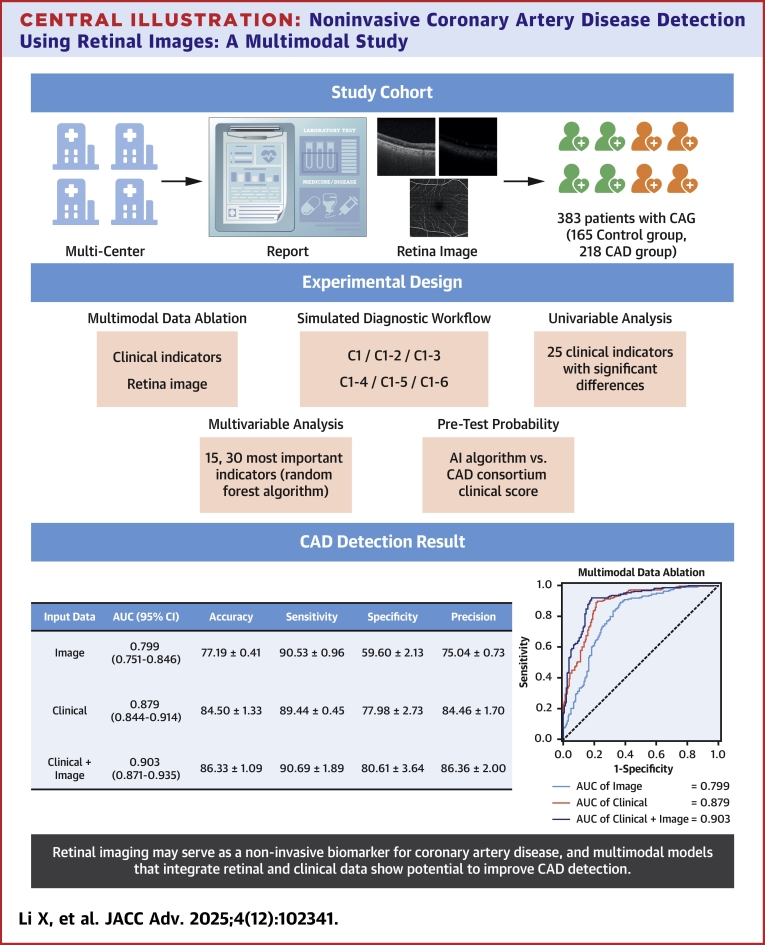
**Noninvasive Coronary Artery Disease Detection Using Retinal Images: A Multimodal Study** AUC = area under the receiver operating characteristic curve; CAD = coronary artery disease; CAG = coronary angiography.

We used gradient-weighted class activation maps^20^ to interpret the role of retinal images in multimodal CAD detection models. Visualization results are shown in Supplemental Figure 6. All retinal images, including those from control and CAD groups, highlight regions of interest under the assumption of CAD. In OCT images from the CAD group, the focus is primarily near the macular fovea, suggesting that clinical features associated with the fovea may serve as risk factors for CAD. Matulevičiūtė et al^21^ also reported that parameters related to the central retinal region can predict CAD, consistent with our findings. Previous studies^22^, ^23^, ^24^, ^25^, ^26^, ^27^ have demonstrated that retinal microvascular abnormalities are directly associated with increased CVD risk. Deep learning methods for extracting detailed information from fundus images to predict CVD risk have been applied.^17^^,^^28^^,^^29^ Ren et al.^30^ constructed a logistic regression model based on parameters of retinal neurovascular structures in OCTA images, achieving an AUC of 0.83 for distinguishing CAD from non-CAD cases. As shown in Supplementary Figure 6, the model focuses on retinal vessels in OCTA images, particularly in regions with denser vasculature on both sides. Similarly, in projection maps, vascular regions are prominently highlighted. Our study suggests that retinal images and retinal vasculature may provide useful information for CAD detection. It should be noted that important features for identifying CAD may vary among individuals.

### Study Limitations

Despite the encouraging results, our study has some limitations. First, we only used OCT, OCTA, and projection maps to construct the vision model. Incorporating additional ophthalmic imaging modalities, such as fundus photography, may further enhance CAD detection capabilities, paving the way for a new paradigm in noninvasive CAD detection research. Second, the data set size was relatively small for deep learning. Although our artificial intelligence model performed well in comparison with the CAD consortium clinical score, its 95% CI was relatively wide. A larger data set would enable the training of more accurate deep learning models and allow for higher-confidence evaluation. Furthermore, the limited sample sizes collected from multiple centers did not allow for the construction of an independent external validation set. This limitation may affect the generalizability and robustness of our conclusions. The insufficient sample size and potential lack of data representativeness could lead to uncertainties in model performance across different clinical settings. Continued data collection from a larger number of clinical centers is needed to build a more extensive and diverse cardio-ophthalmic data set and enable future external validation. In addition, we excluded some patients, such as those with diabetic retinopathy or a history of retinal surgery, to minimize confounding effects on retinal imaging and ensure a more consistent dataset for model development. However, this exclusion may limit the model's generalizability to broader clinical populations. Future studies should aim to include more diverse patient cohorts to validate the model's performance across real-world clinical scenarios.

## Conclusions

In conclusion, our findings suggest that retinal images hold promise as noninvasive biomarkers for CAD detection. The incorporation of retinal images into multimodal detection models provided complementary value to clinical indicators, leading to improvements in accuracy and sensitivity in certain settings. Although not all comparisons reached statistical significance, the results highlight the potential role of retinal imaging in supporting CAD risk assessment, particularly in enhancing sensitivity for patient identification. Future research should focus on large-scale validation, exploration of clinical applicability, and the development of multimodal CAD detection frameworks that integrate noninvasive factors to improve generalizability and real-world utility. In addition, prospective studies are warranted to evaluate the feasibility of deploying such models in real-time clinical decision support.

Perspectives **COMPETENCY IN MEDICAL KNOWLEDGE:** This study demonstrates that retinal images may serve as valuable noninvasive biomarkers for CAD detection. Multimodal CAD detection models can incorporate retinal imaging information to capture risk factors that may not be readily discernible from clinical indicators alone. In classifying CAD cases, the inclusion of retinal images improved performance in several experimental settings, with some improvements reaching statistical significance. These findings suggest that retinal imaging may add prognostic value to simple baseline clinical models, particularly in earlier, less information-rich stages of diagnosis.

**TRANSLATIONAL OUTLOOK:** Our findings provide a quantitative estimation of the diagnostic value retinal imaging may add to existing clinical tools for CAD detection. Although we acknowledge that retinal imaging requires specialized equipment and may not yet be as accessible or cost-effective as traditional tests such as ECGs or laboratory assays, the study offers a foundation for further exploration. Future research should focus on validating these results in larger and more diverse populations, and on determining the most appropriate clinical scenarios where retinal imaging could offer complementary benefits. In addition, developing deployable multimodal models that integrate retinal images and clinical data, supported by real-time inference systems, could contribute to more accurate risk stratification, earlier intervention, and more personalized cardiovascular care. We believe that this work may serve as a reference point for subsequent investigations, helping to clarify the potential role of retinal imaging in noninvasive CAD screening pathways.

## Funding support and author disclosures

This study was supported, in whole or in part, by the Major Research Plan of the 10.13039/501100014857National Natural Science Foundation of China (92370109); the 10.13039/501100014857National Natural Science Foundation of China (62172223, 82170269); 10.13039/501100012226Natural Science Foundation of Jiangsu Province (BK20252033); the 2024 Changzhou City's Eleventh Batch of Science and Technology Plan Projects (Applied Basic Research Special Project - Health and Medical Field, Social Capital Funded Project, CJ20244001); and the Medical Research Project of the 10.13039/100017962Jiangsu Provincial Health Commission (K2024009). The authors have reported that they have no relationships relevant to the contents of this paper to disclose.

## Supplementary Material

Supplemental Appendi x

## References

[1] Schutte A.E., Srinivasapura Venkateshmurthy N., Mohan S., Prabhakaran D. (2021). Hypertension in low- and middle-income countries. Circ Res.

[2] Timmis A., Vardas P., Townsend N. (2022). European society of cardiology: cardiovascular disease statistics 2021. Eur Heart J.

[3] Tsao C.W., Aday A.W., Almarzooq Z.I. (2023). Heart disease and stroke statistics—2023 update: a report from the American Heart Association. Circulation.

[4] Labrecque Langlais É., Corbin D., Tastet O. (2024). Evaluation of stenoses using AI video models applied to coronary angiography. NPJ Digit Med.

[5] Montalescot G., Sechtem U., Achenbach S. (2013). 2013 ESC guidelines on the management of stable coronary artery disease: the task force on the management of stable coronary artery disease of the European society of cardiology. Eur Heart J.

[6] Jolly S.S., Amlani S., Hamon M., Yusuf S., Mehta S.R. (2009). Radial versus femoral access for coronary angiography or intervention and the impact on major bleeding and ischemic events: a systematic review and meta-analysis of randomized trials. Am Heart J.

[7] Borren N., Maas A.H., Ottervanger J.P. (2015). Stop invasive coronary angiography as the gold standard for the diagnosis of stable angina!. Intervent Cardiol.

[8] Wong T.Y., Mitchell P. (2004). Hypertensive retinopathy. N Engl J Med.

[9] Flammer J., Konieczka K., Bruno R.M., Virdis A., Flammer A.J., Taddei S. (2013). The eye and the heart. Eur Heart J.

[10] Tseng R.M.W.W., Rim T.H., Shantsila E. (2023). Validation of a deep-learning-based retinal biomarker (Reti-CVD) in the prediction of cardiovascular disease: data from UK Biobank. BMC Med.

[11] Cheung C.Y., Xu D., Cheng C.-Y. (2020). A deep-learning system for the assessment of cardiovascular disease risk via the measurement of retinal-vessel calibre. Nat Biomed Eng.

[12] McGeechan K. (2009). Meta-analysis: retinal vessel caliber and risk for coronary heart disease. Ann Intern Med.

[13] Gopinath B., Chiha J., Plant A.J.H. (2014). Associations between retinal microvascular structure and the severity and extent of coronary artery disease. Atherosclerosis.

[14] Ahmad M., Kaszubski P.A., Cobbs L., Reynolds H., Smith R.T. (2017). Choroidal thickness in patients with coronary artery disease. PLOS One.

[15] Theuerle J.D., Al-Fiadh A.H., Amirul Islam F.M. (2021). Impaired retinal microvascular function predicts long-term adverse events in patients with cardiovascular disease. Cardiovasc Res.

[16] Lin S., Li Z., Fu B. (2020). Feasibility of using deep learning to detect coronary artery disease based on facial photo. Eur Heart J.

[17] Poplin R., Varadarajan A.V., Blumer K. (2018). Prediction of cardiovascular risk factors from retinal fundus photographs via deep learning. Nat Biomed Eng.

[18] Lee Y.C., Cha J., Shim I. (2023). Multimodal deep learning of fundus abnormalities and traditional risk factors for cardiovascular risk prediction. npj Digit Med.

[19] Al-Zaiti S.S., Martin-Gill C., Zègre-Hemsey J.K. (2023). Machine learning for ECG diagnosis and risk stratification of occlusion myocardial infarction. Nat Med.

[20] Selvaraju R.R., Cogswell M., Das A., Vedantam R., Parikh D., Batra D. (2017). 2017 IEEE International Conference on Computer Vision (ICCV).

[21] Matulevičiūtė I., Sidaraitė A., Tatarūnas V., Veikutienė A., Dobilienė O., Žaliūnienė D. (2022). Retinal and choroidal thinning—a predictor of coronary artery occlusion?. Diagnostics.

[22] Wong T.Y., Klein R., Couper D.J. (2001). Retinal microvascular abnormalities and incident stroke: the atherosclerosis risk in communities study. Lancet.

[23] Campbell M.D., Laitinen T.T., Hughes A. (2018). Impact of ideal cardiovascular health in childhood on the retinal microvasculature in midadulthood: cardiovascular risk in young Finns study. J Am Heart Assoc.

[24] Ding J., Wai K.L., McGeechan K. (2014). Retinal vascular caliber and the development of hypertension: a meta-analysis of individual participant data. J Hypertens.

[25] Owen C.G., Rudnicka A.R., Welikala R.A. (2019). Retinal vasculometry associations with cardiometabolic risk factors in the European prospective investigation of cancer—Norfolk study. Ophthalmology.

[26] Seidelmann S.B., Claggett B., Bravo P.E. (2016). Retinal vessel calibers in predicting long-term cardiovascular outcomes: the atherosclerosis risk in communities study. Circulation.

[27] Wang J., Jiang J., Zhang Y., Qian Y.W., Zhang J.F., Wang Z.L. (2019). Retinal and choroidal vascular changes in coronary heart disease: an optical coherence tomography angiography study. Biomed Opt Express.

[28] Rim T.H., Lee C.J., Tham Y.-C. (2021). Deep-learning-based cardiovascular risk stratification using coronary artery calcium scores predicted from retinal photographs. Lancet Digit Health.

[29] Ting D.S.W., Wong T.Y. (2018). Eyeing cardiovascular risk factors. Nat Biomed Eng.

[30] Ren Y., Hu Y., Li C. (2023). Impaired retinal microcirculation in patients with non-obstructive coronary artery disease. Microvasc Res.

