# Supplementary material for: Noninvasive Coronary Artery Disease Detection Using Retinal Images: A Multimodal Study
Source: JACC Adv. 2025 Nov 19;4(12):102341. doi: 10.1016/j.jacadv.2025.102341 (PMC12670101; doi:10.1016/j.jacadv.2025.102341)
Supplement: Supplemental Appendi — x [file mmc1.pdf]

Supplemental Appendix

Table of Contents

eMethods

eData Processing and Model Architecture……………………………………………2
eFigure 1. Clinical information and image data preprocessing flow………………………5
eTable 1. Clinical characteristics…………………………………………………..……6
eFigure 2. Multimodal CAD Detection Model……………………………………………9
eFigure 3. Clinical Indicator Processing and Feature Extraction…………………………10
eTable 2. Clinical indicators used in Univariable, Multivariable Analysis, and Pre-Test Probability ……….……………………………………………………………………11

eResult

eFigure 4. Confusion matrices for all algorithms…………………………………..……12
eFigure 5. Confusion matrices for all algorithms in Pre-Test Probability experiments……13
eFigure 6. Class Activation Maps of Retinal Images………………………………………14

eFigure 7. Presentation of retinal images of excluded patients……………………………15
eTable 3. Statistics on the number of missing values in clinical indicators………………….....16

eReferences……………………………………………………………..……………………17

eData Processing and Model Architecture

Data Collection

Baseline interviews were conducted to collect data on lifestyle factors (e.g., smoking), clinical pain symptoms, medical history, and medications. Additional demographic characteristics, risk factors, laboratory test, and electrocardiographic were extracted from medical records post-procedure. Standardized imaging protocols were employed to perform three-dimensional OCT imaging of the macular region in both eyes of each patient. Imaging systems differed across participating centers: The First Affiliated Hospital of Nanjing Medical University and the Affiliated Hospital of Yangzhou University used the AngioVue OCT systems (Wavelength: 840 nm, Version A2018.1.1.63, Optovue, CA) with a field of view of 6 mm * 6 mm and a depth resolution of 3.125 µm/pixel. The OCT/OCTA volume size was 640*400*400. Qijiang People's Hospital of Chongqing used YG-100K PRO OCT systems (Wavelength: 1060 nm, Version 1.3.3.1.1, TowardPi, CN) with a field of view of 12 mm * 12 mm and a depth resolution of 2.819 µm/pixel. The OCT volume size was 1064*768*768. Liyang People's Hospital used YG-100K PRO OCT systems (Wavelength: 1060 nm, Version 1.3.3.1, TowardPi, CN) with a field of view of 6 mm * 6 mm and a depth resolution of 6.138 µm/pixel. The OCT volume size was 522*256*512.

Labeling and Retinal Image Preprocessing

All enrolled patients were categorized into a control group and a CAD group. CAD was defined as more than 50% narrowing in any major coronary artery, including the left main (LM), left anterior descending (LAD), left circumflex (LCX), or right coronary artery (RCA), as determined by CAG.[1-3] Two radiologists, blinded to the study design, independently reviewed each patient's CAG results to assess the degree of coronary stenosis. In cases of disagreement, a third radiologist reviewed the results to reach a consensus. The quality of OCT/OCTA images was evaluated by two experienced ophthalmologists. Unqualified images, such as those with incomplete retinal structure or poor resolution, were excluded from the final analysis (see eFigure 7), with the processing flow shown in eFigure 1. OCT volumetric data were resampled to a unified format of 640*400*400, with a field of view of 6 mm * 6 mm and a scan depth of 2 mm. For retinal OCT data obtained using TowardPi devices, the Neural Preset[4] method was applied to adapt the style to match Optovue data. Additionally, OCT data were processed using the Iowa software (OCTExplorer 3.8)[5] to perform retinal layer segmentation and extract the inner limiting membrane (upper boundary) and Bruch's membrane (lower boundary). Using this boundary information, OCTA data were restricted to projection, generating OCTA projection maps.

Missing Data Handling

During the collection of clinical data, varying degrees of missing information were observed. For continuous variables such as weight, cholesterol levels, and fasting blood glucose, statistical means were used for group filling. For discrete variables such as ST segment change, pathologic Q waves and T wave inversion, statistical mode filling was utilized. Variables with extensive missing clinical indicators were excluded from the analysis (lipoprotein(a), hemoglobin A1c, fibrinogen). The clinical indicators with missing values and the corresponding number of missing entries are listed in eTable 3. The final set of clinical indicators used in the study (45 variables) is detailed in eTable 1. For imaging data, some patients only had OCT data without accompanying OCTA data. Given that OCTA-derived

blood flow information is closely associated with CAD, we leveraged our previous work[6] to generate OCTA data based on OCT scans (see eFigure 1).

Model Architecture

The multimodal coronary artery disease (CAD) detection model is illustrated in eFigure 2. For retinal images, three identical ResNet34 networks[7] are employed to extract features from OCT, OCTA, and Projection Map images, respectively. The feature dimensions for OCT and OCTA are both 128, and their concatenation results in a 256-dimensional feature vector. The feature dimension for the Projection Map is 256. For clinical information, two models are used to extract features, as detailed in eFigure 3. On one hand, numerical clinical data are processed by a multilayer perception (MLP) to extract a 256-dimensional feature vector. On the other hand, numerical data are transformed into textual descriptions incorporating their clinical significance. These textual descriptions are then fed into the BiomedBERT model to extract 256-dimensional text features. BiomedBERT, developed by Gu et al.[8], is a foundational biomedical language model trained on PubMed abstracts and full-text articles from PubMed Central. After feature extraction, a cross-modal data attention module is utilized to enable information exchange between different modalities, fully leveraging the integrated features to enhance CAD detection performance. Finally, the fused multimodal features are aggregated and passed through a classifier to produce the classification output. Additionally, by modifying the feature extraction section and adjusting the input dimension of the classifier, the multimodal model in eFigure 2 can be decomposed into two unimodal models (using only retinal images and only clinical indicators) without altering the classifier structure.

Loss Function

Two loss functions are applied during the training of the multimodal CAD detection model. The supervised classification loss is calculated using cross-entropy:

$$L_{CE} = -\sum(y_i * log(p_i) + (1 - y_i) * log(1 - p_i))$$

where y_i represents the ground truth label for the i-th sample, given by clinicians, with values of 0 (normal) or 1 (CAD), and p_i denotes the model-predicted probability of CAD for the i-th sample.

In addition, mutual information loss, computed using the Mutual Information Neural Estimation (MINE) method[9], is employed as an auxiliary loss to ensure consistency among multimodal features. MINE uses a differentiable neural network to estimate the joint and marginal probability distributions of features. The mutual information (MI) loss is defined as:

$$L_{MI} = -(\sum T(x_i, y_i) - log\sum e^{T(x_j, y_j^-)})$$

where $T(x, y)$ is the neural network output score, (x_i, y_i) are positive sample pairs, and (x_j, y_j^-) are negative sample pairs independently drawn from the marginal distributions.

The total loss is defined as:

$$L = L_{CE} + \lambda L_{MI}$$

Training Protocols

All networks in this study were implemented using the PyTorch deep learning framework and executed on a single NVIDIA RTX 4090 GPU. The input size for OCT, OCTA, and Projection Map images were 400*400, while the input dimensions for clinical indicators varied dynamically depending on the experiment. The MLP consists of three LBR blocks followed by a linear layer. Each LBR block comprises a linear layer, batch normalization, and ReLU activation. The classifier includes two LBR blocks and a linear layer. ResNet34 uses pre-trained weights, with gradients updated during training.

BiomedBERT parameters remain frozen and are not updated. Other network parameters are initialized using the Kaiming distribution. The models were trained for 100 epochs with a batch size of 16. All parameters were optimized using the SGD algorithm with an initial learning rate of 1e-3, a momentum of 0.9, and a weight decay of 5e-4. StepLR was used for dynamic learning rate adjustment with a step size of 20 and a decay factor of 0.8.

Model Evaluation

The control group (non-CAD patients) was labeled as negative, while the CAD group was labeled as positive. The evaluation metrics include accuracy, precision (Positive Predictive Value, PPV), sensitivity, specificity, F1-score, and negative predictive value (NPV): Accuracy represents the proportion of correctly classified samples. Precision/PPV measures the proportion of true positives among all predicted positives. Sensitivity is the proportion of true positives among all actual positives. Specificity is the proportion of true negatives among all actual negatives. F1-score is the harmonic mean of precision and sensitivity, balancing model performance in cases of imbalanced datasets. NPV measures the proportion of true negatives among all predicted negatives. The ROC curve plots sensitivity against 1−Specificity, describing the trade-off between true positive and false positive rates as the classification threshold varies. Formulas for evaluation metrics:

$$Accuracy = \frac{TP + TN}{TP + FP + FN + TN}$$

$$Precision/PPV = \frac{TP}{TP + FP}$$

$$Sensitivity = \frac{TP}{TP + FN}$$

$$Specificity = \frac{TN}{TN + FP}$$

$$F1 - score = \frac{2 * Precision * Sensitivity}{Precision + Sensitivity}$$

$$NPV = \frac{TN}{TN + FN}$$

Here, True Positives (TP) are correctly classified positive samples, True Negatives (TN) are correctly classified negative samples, False Positives (FP) are misclassified negative samples predicted as positive, and False Negatives (FN) are misclassified positive samples predicted as negative.

eFigure 1. Clinical information and image data preprocessing flow

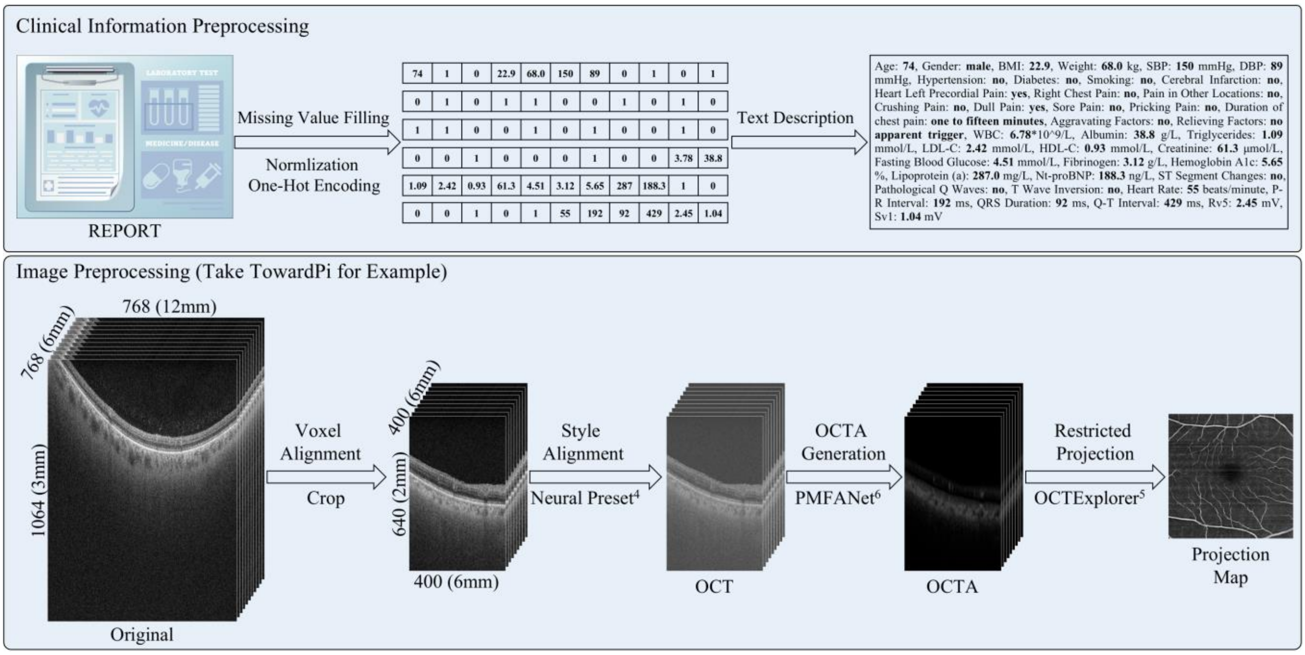

eTable 1. Clinical characteristics and pain symptoms

	Control Group (n=165)	CAD Group (n=218)	P-value
Baseline			
Age (years) [b]	61.81 ± 11.42 (25-82)	63.44 ± 10.81 (31-83)	0.23
Male, n (%) [c]	76 (46.06)	153 (70.18)	**<0.001**
Weight (kg) [b]	67.43 ± 12.64 (40-120)	68.12 ± 11. 30 (21-105)	0.16
BMI (kg/m^2) [b]	25.12 ± 3.73 (16.9-41.6)	24.88 ± 3.43 (17.9-50)	0.81
SBP (mmHg)[a]	131.01 ± 18.18 (82-195)	130.40 ± 16.62 (91-76)	0.73
DBP (mmHg)[b]	79.93 ± 14.36 (50-168)	79.44 ± 11.10 (55-121)	0.95
Medical history			
Hypertension, n (%) [c]	78 (47.27)	157 (72.02)	**<0.001**
Diabetes mellitus, n (%) [c]	29 (17.58)	69 (31.65)	**0.003**
Smoking, n (%) [c]	21 (12.73)	74 (33.94)	**<0.001**
Cerebral infarction, n (%) [c]	9 (5.45)	16 (9.70)	0.60
Heart failure, n (%) [c]	3 (1.82)	1 (0.46)	0.43
Medications			
β-blockers, n (%) [c]	65 (39.39)	137 (62.84)	**<0.001**
ACEI or ARB, n (%) [c]	38 (23.03)	56 (25.69)	0.63
MRA, n (%) [c]	10 (6.06)	13 (5.96)	1.00
Diuretics, n (%) [c]	33 (20.00)	21 (9.63)	**0.006**
CCB, n (%) [c]	50 (30.30)	73 (33.49)	0.58
Antiplatelet, n (%) [c]	71 (43.03)	182 (83.49)	**<0.001**
Anticoagulant, n (%) [c]	33 (20.00)	93 (42.66)	**<0.001**
Statin, n (%) [c]	120 (72.73)	215 (98.62)	**<0.001**
Insulin, n (%) [c]	1 (0.61)	12 (5.50)	**0.019**
Oral hypoglycemic, n (%) [c]	24 (14.55)	54 (24.77)	**0.020**
GLP-1, n (%) [c]	1 (0.61)	4 (1.83)	0.55
Laboratory test			
WBC (10^9/L) [b]	6.21 ± 1.74 (3.31-12.46)	6.64 ± 2.04 (2.55-14.54)	**0.035**
ALB (g/L) [b]	40.90 ± 3.51 (32.1-54.3)	39.49 ± 4.74 (3.06-71.3)	**<0.001**
TC (mmol/L) [b]	4.39 ± 1.01 (2.2-9.25)	3.87 ± 1.00 (1.43-8.88)	**<0.001**
TG (mmol/L) [b]	1.82 ± 1.15 (0.36-6.42)	1.71 ± 1.37 (0.43-15.07)	0.13
LDL-C (mmol/L) [b]	2.61 ± 0.92 (1.03-7.29)	2.34 ± 0.73 (0.65-4.45)	**0.003**
HDL-C (mmol/L) [b]	1.20 ± 0.31 (0.61-2.59)	1.03 ± 0.26 (0.55-2.23)	**<0.001**
Creatinine (umol/L) [b]	71.38 ± 31.93 (39-393.8)	73.34 ± 17.58 (40-195.3)	**0.013**
FBG (mmol/L) [b]	5.51 ± 1.55 (3.22-13.47)	5.45 ± 1.87 (2.31-16.97)	0.10
Electrocardiography			
HR (times/minute) [b]	70.98 ± 15.37 (48-185)	71.46 ± 12.11 (43-116)	0.23
P-R (ms)[b]	163.78 ± 22.34 (117-256)	166.30 ± 28.29 (0-272)	0.23
QRS (ms)[b]	95.88 ± 16.07 (59-177)	96.578 ± 16.61 (48-174)	0.69
Q-T (ms)[b]	399.43 ± 40.07 (222-549)	399.76 ± 39.90 (117-513)	0.99

RV5 (mV)[b]	1.45 ± 0.56 (0.24-3.42)	1.41 ± 0.61 (0.03-3.8)	0.43
SV1 (mV)[b]	0.73 ± 0.51 (0-3.661)	0.75 ± 0.48 (0-3.269)	0.60
RV5+SV1 (mV)[b]	2.18 ± 0.86 (0.5-6.51)	2.16 ± 0.81 (0.46-4.7)	0.90
ST Segment Changes, n (%) [c]	17 (10.30) / 13 (7.88) / 135 (81.82)	16 (7.34) / 36 (16.51) / 166 (76.15)	**0.033**
Pathological Q waves, n (%) [c]	3 (1.82)	48 (22.02)	**<0.001**
T-wave inversion, n (%) [c]	61 (36.97)	113 (51.83)	**0.005**
Chest Pain and Radiation Sites			
Left Precordial, n (%) [c]	39 (23.64)	119 (54.59)	**<0.001**
Right Chest, n (%) [c]	3 (1.82)	4 (1.83)	1.00
Left Shoulder, n (%) [c]	3 (1.82)	12 (5.50)	0.12
Left Upper Arm, n (%) [c]	1 (0.61)	5 (2.29)	0.37
Neck, n (%) [c]	2 (1.21)	3 (1.38)	1.00
Back, n (%) [c]	6 (3.64)	19 (8.72)	0.074
Abdominal, n (%) [c]	1 (0.61)	1 (0.46)	1.00
Other Locations, n (%) [c]	1 (0.61)	4 (1.83)	0.94
Nature of Chest Pain			
Crushing Pain, n (%) [c]	9 (5.45)	55 (25.23)	**<0.001**
Dull Pain, n (%) [c]	24 (14.55)	43 (19.72)	0.24
Sore Pain, n (%) [c]	0 (0.00)	4 (1.83)	0.21
Burning Pain, n (%) [c]	1 (0.61)	7 (3.21)	0.16
Stabbing Pain, n (%) [c]	0 (0.00)	1 (0.46)	1.00
Pricking Pain, n (%) [c]	6 (3.64)	14 (6.42)	0.33
Duration of Chest Pain, n (%) [c]	119 (72.12) / 7 (4.25) / 26 (15.76) / 4 (2.42) / 9 (5.45)	91 (41.74) / 7 (3.21) / 74 (33.94) / 19 (8.72) / 27 (12.38)	**<0.001**
Aggravating Factors, n (%) [c]	119 (72.12) / 32 (19.40)/ 4 (2.42) / 10 (6.06)	91 (41.74) / 67 (30.74) / 21 (9.63) / 39 (17.89)	**<0.001**
Relieving Factors, n (%) [c]	119 (72.12) / 43 (26.06) / 1 (0.61) / 2 (1.21) / 0 (0.00)	91 (41.74) / 113 (51.84) / 0 (0.00) / 9 (4.13) / 5 (2.29)	**<0.001**

Statistical results are expressed as mean ± standard deviation (min-max) or n (%).
ACRI/ARB = angiotensin-converting enzyme inhibitors or II receptor blockers, ALB = albumin,
BMI = body mass index, CCB = calcium channel blockers, DBP = diastolic blood pressure, FPG = fasting blood glucose, GLP-1 = glucagon-like peptide-1, HDL-C = high-density lipoprotein cholesterol, HR = heart rate, LDL-C = low-density lipoprotein cholesterol, MRA = mineralocorticoid receptor antagonists, P-R = P-R interval, QRS = QRS duration, Q-T = QT interval, RV5 = R-wave amplitude in lead V5, SBP = systolic blood pressure, SV1 = S-wave amplitude in lead V1, TC = total cholesterol, TG = triglycerides, WBC = white blood count.
ST Segment Changes = depression / elevation / no. The ST segment changes were defined as an elevation of ≥ 0.1 mV or a depression of ≥ 0.05 mV in at least two contiguous leads
Duration of Chest Pain = none (without chest pain) / < 1 minute / 1-15 minutes / 15-30 minutes / >30 minutes
Aggravating Factors = none (without chest pain) / no / During activity / After activity
Relieving Factors = none (without chest pain) / no apparent trigger / < 1 minute / 1-15 minutes / > 15 minutes after nitroglycerin or quick-acting coronary pills
n = number of patients

A p-value <0.05 is considered statistically significant

a = Student t-test

b = Mann-Whitney U test

c = Chi-squared test

eFigure 2. Multimodal CAD Detection Model

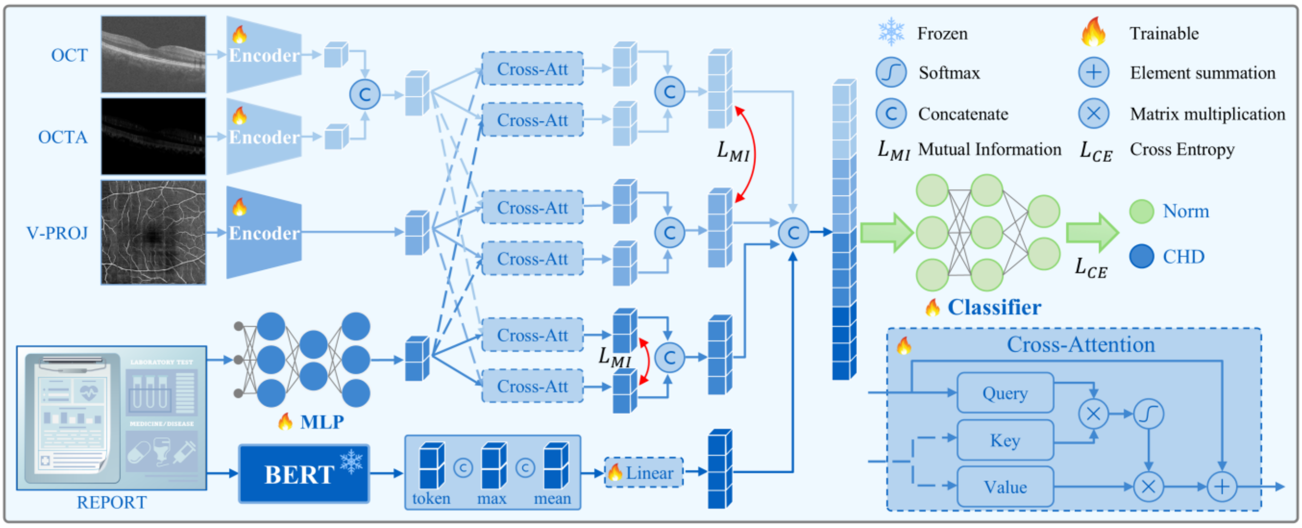

eFigure 3. Clinical Indicator Processing and Feature Extraction

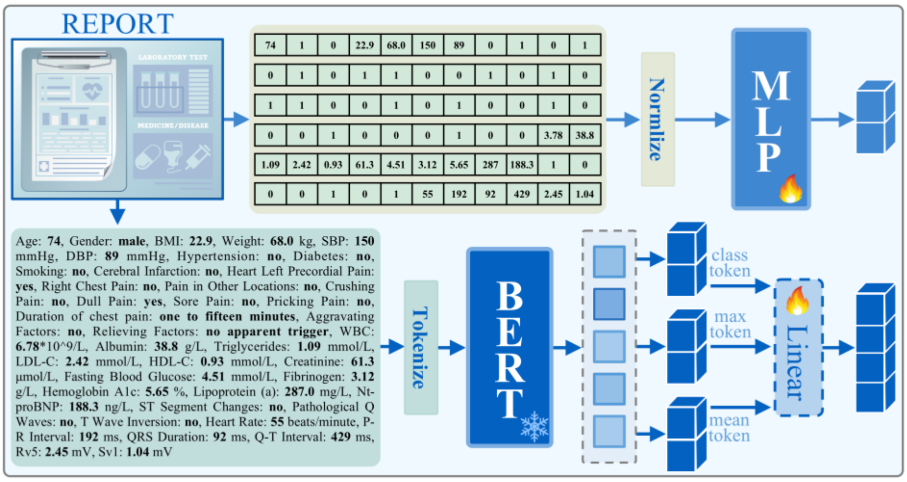

Numerical data from patient records are normalized and one-hot encoded to form a one-dimensional vector, which is processed by an MLP to extract numerical features. The numerical data are also embedded into a template to form complete textual descriptions, which are processed by BiomedBERT to extract class token, max token, and mean token features that are concatenated into the final text feature vector.

eTable 2. Clinical indicators used in Univariate, Multivariate Analysis, and Pre-Test Probability

Univariable Analysis (25)	Multivariable Analysis (Top30)	Pre-Test Probability
Gender	HDL-C (mmol/L)	Age (years)
Hypertension	TC (mmol/L)	Gender
Diabetes mellitus	Antiplatelet	Hypertension
Smoking	Anticoagulants	Diabetes mellitus
β-blockers	Statins	Smoking
Diuretics	Heart Rate	TC (mmol/L)
Antiplatelet	TG (mmol/L)	TG (mmol/L)
Anticoagulant	LDL-C (mmol/L)	LDL-C (mmol/L)
Statin	FBG (mmol/L)	HDL-C (mmol/L)
Insulin	Sv1 (ms)	Left Precordial
Oral hypoglycemic	ALB (g/L)	Right Chest
WBC (10^9/L)	Creatinine (umol/L)	Left Shoulder
ALB (g/L)	BMI (kg/m^2)	Left Upper Arm
TC (mmol/L)	Q-T (ms)	Neck
LDL-C (mmol/L)	Age (years)	Back
HDL-C (mmol/L)	WBC (10^9/L)	Abdominal
Creatinine (umol/L)	Rv5+Sv1 (ms)	Other Locations
ST Segment Changes	DBP (mmHg)	Crushing Pain
Pathologic Q waves	Rv5 (ms)	Dull Pain
T-wave inversion	SBP (mmHg)	Sore Pain
Left Precordial	Duration of Chest Pain	Burning Pain
Crushing Pain	Weight (kg)	Stabbing Pain
Duration of Chest Pain	P-R (ms)	Pricking Pain
Aggravating Factors	Hypertension	Duration of Chest Pain
Relieving Factors	QRS (ms)	Aggravating Factors
	Pathologic Q waves	Relieving Factors
	Left Precordial	
	Relieving factors	
	Aggravating factors	
	Crushing Pain	

Indicators for multivariable analysis are ranked by importance in descending order based on a random forest model.

eFigure 4. Confusion matrices for all algorithms

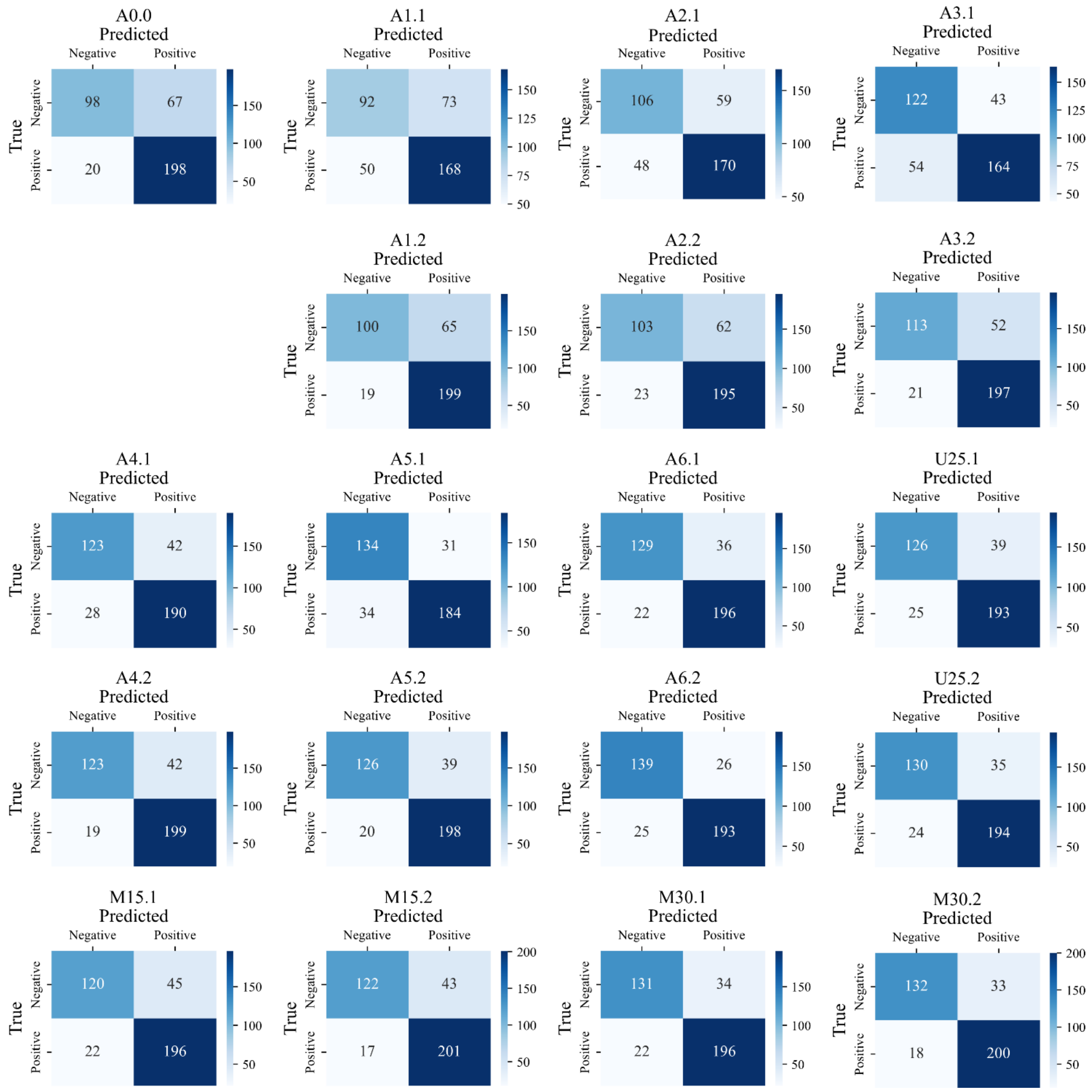

Multimodal Data Ablation, Simulated Diagnostic Workflow, Univariable Analysis, and Multivariable Analysis. The confusion matrices presented correspond to the median accuracy trial among three repeated experiments, thus slight deviations may exist compared to tabulated metrics (mean ± standard deviation).

eFigure 5. Confusion matrices for all algorithms in Pre-Test Probability experiments

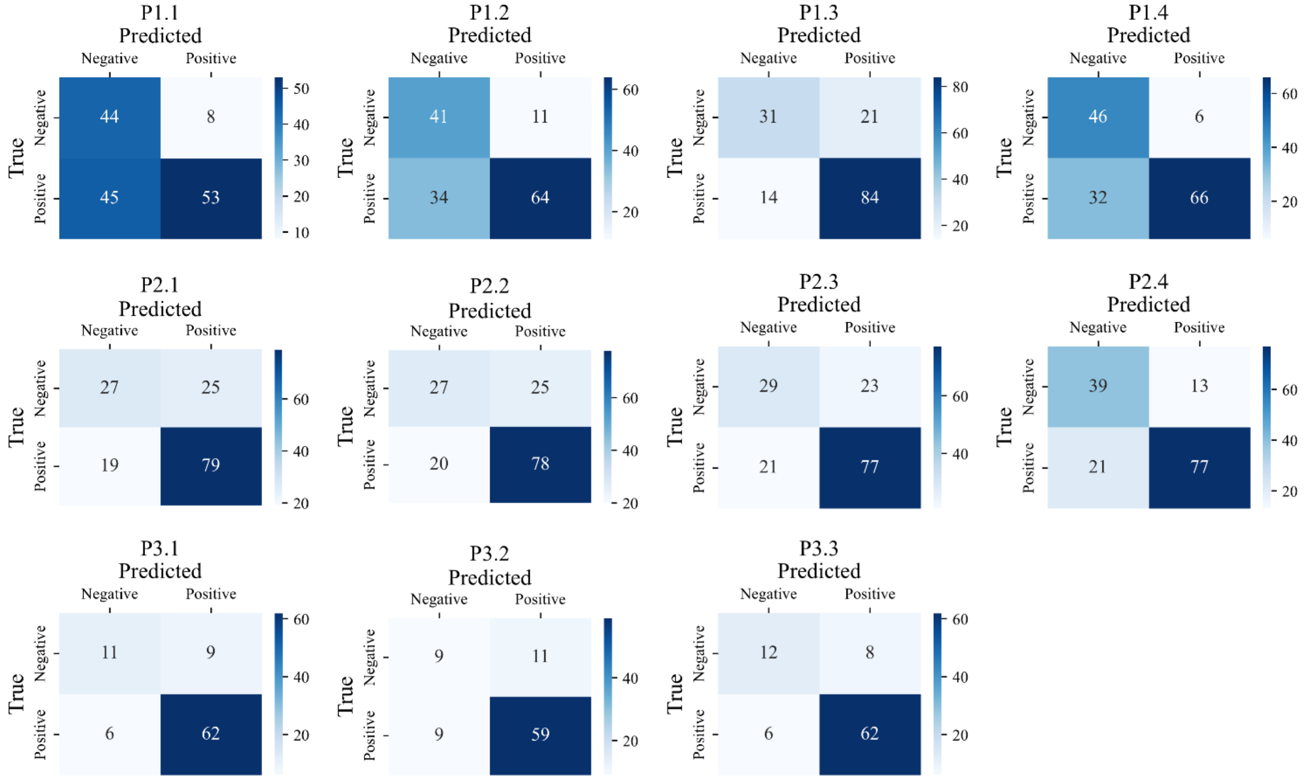

Described similarly to **eFigure 4.**

eFigure 6. Class Activation Maps of Retinal Images

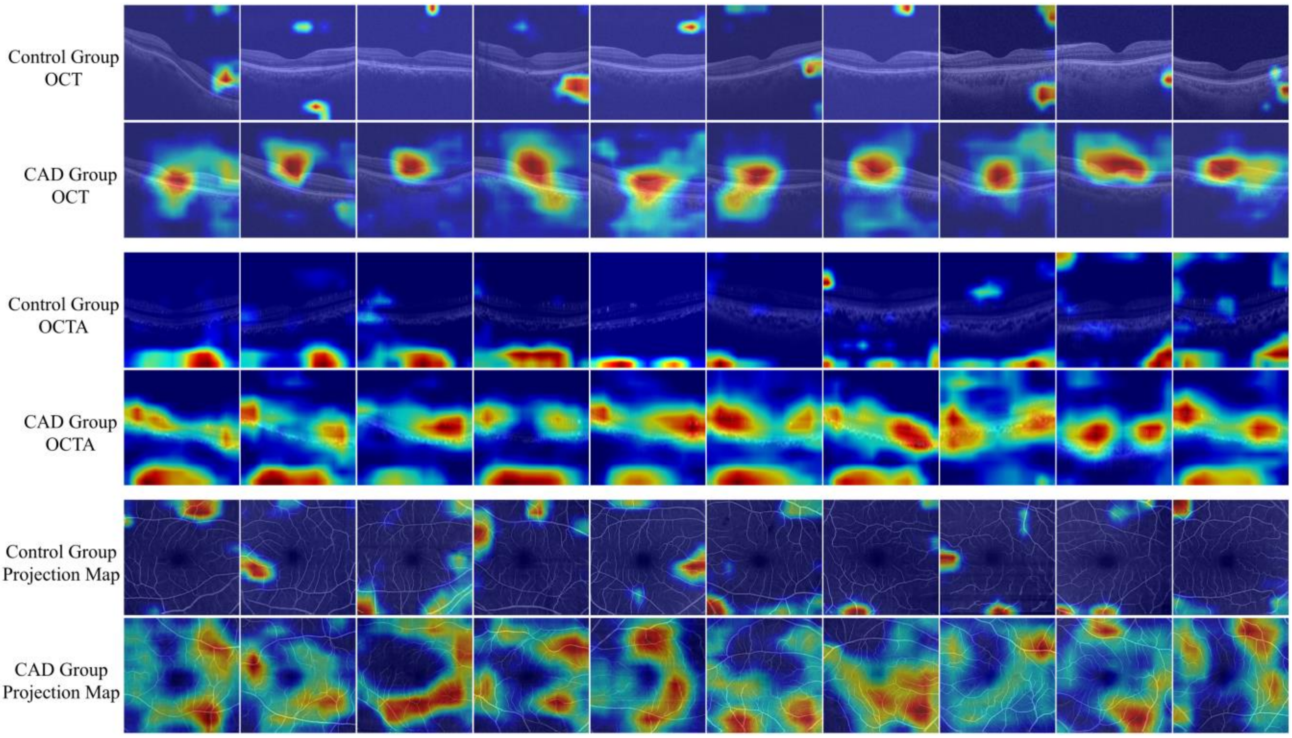

CAMs are shown for both the control group and the CAD group, highlighting the regions of interest under the condition that the model identifies the sample as coronary artery disease. Areas closer to red in the image indicate areas of greater interest to the network.

eFigure 7. Presentation of retinal images of excluded patients

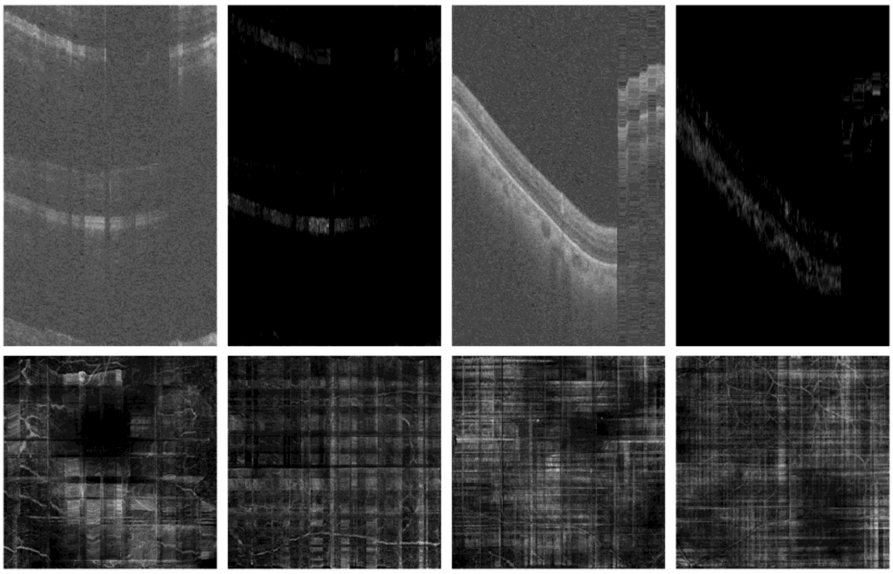

The OCT and OCTA images exhibited incomplete or disrupted retinal structures, while the projection maps were disorganized, making it impossible to identify the retinal vasculature. A total of 75 patients were excluded based on these criteria.

eTable 3. Statistics on the number of missing values in clinical indicators

Clinical Indicators	Number
Weight (kg)	6
BMI (kg/m^2)	11
WBC (10^9/L)	6
ALB (g/L)	2
TC (mmol/L)	34
TG (mmol/L)	3
LDL-C (mmol/L)	4
HDL-C (mmol/L)	4
Creatinine (umol/L)	2
FBG (mmol/L)	8
HR (times/minute)	1
P-R (ms)	12
SV1 (mV)	1
RV5+SV1 (mV)	1
ST Segment Changes	2
Pathological Q waves	2
T-wave inversion	3
Lp(a) (g/L) [a]	134
HbA1c (%) [a]	102
FIB (g/L) [a]	117

No patients were excluded based on clinical indicators. Abbreviation same as eTable 1.
Lp(a) = lipoprotein (a), HbA1c = hemoglobin A1c, FIB = fibrinogen.
a = Excluded from the analysis

eReferences

1. Diamond GA, Forrester JS. Analysis of probability as an aid in the clinical diagnosis of coronary-artery disease. N Engl J Med. 1979;300:1350–8.

2. Graham IM. Diagnosing coronary artery disease--the diamond and forrester model revisited. Eur Heart J. 2011;32:1311–2.

3. Bittencourt MS, Hulten E, Polonsky TS, Hoffman U, Nasir K, Abbara S, et al. European society of cardiology–recommended coronary artery disease consortium pretest probability scores more accurately predict obstructive coronary disease and cardiovascular events than the diamond and forrester score: the partners registry. Circulation. 2016;134:201–11.

4. Ke Z, Liu Y, Zhu L, Zhao N, Lau RWH. Neural preset for color style transfer. In: 2023 IEEE/CVF Conference on Computer Vision and Pattern Recognition (CVPR). Vancouver, BC, Canada: IEEE; 2023. p. 14173–82.

5. Garvin MK, Abramoff MD, Xiaodong Wu, Russell SR, Burns TL, Sonka M. Automated 3-D Intraretinal Layer Segmentation of Macular Spectral-Domain Optical Coherence Tomography Images. IEEE Trans Med Imag. 2009;28:1436–47.

6. Huang K, Su N, Tao Y, Li M, Ma X, Ji Z, et al. Cross-device OCTA generation by patch-based 3D multi-scale feature adaption. IEE Trans Emerg Topics Comput Intell. 2024;8:641–53.

7. He K, Zhang X, Ren S, Sun J. Deep Residual Learning for Image Recognition. In: 2016 IEEE Conference on Computer Vision and Pattern Recognition (CVPR). Las Vegas, NV, USA: IEEE; 2016. p. 770–8.

8. Gu Y, Tinn R, Cheng H, Lucas M, Usuyama N, Liu X, et al. Domain-Specific Language Model Pretraining for Biomedical Natural Language Processing. ACM Trans Comput Healthcare. 2022;3:1–23.

9. Belghazi MI, Baratin A, Rajeswar S, Ozair S, Bengio Y, Courville A, et al. MINE: mutual information neural estimation. In: Proceedings of the 35th International Conference on Machine Learning. PMLR; 2018. p. 531–40.
